# Re-examination of chimp protein kinases suggests "novel architectures" are gene prediction artifacts

**DOI:** 10.1186/1471-2164-11-66

**Published:** 2010-01-27

**Authors:** Keith Robison

**Affiliations:** 1 Omics! Omics!, 73 Tewksbury St, Andover MA 01810, USA; 2 Current address: Infinity Pharmaceuticals Inc. 780 Memorial Drive. Cambridge MA 02139, USA

## Abstract

**Background:**

Anamika *et al*[1] recently published in this journal a sequence alignment analysis of protein kinases encoded by the chimpanzee genome in comparison to those in the human genome. From this analysis they concluded that several chimpanzee kinases have unusual domain arrangements.

**Results:**

Re-examination of these kinases reveals claimed novel arrangements cannot withstand scrutiny; each is either not novel or represents over-analysis of weakly confident computer generated gene models. Additional sequence evidence available at the time of the paper's submission either directly contradict the gene models or suggest alternate gene models. These alternate models would minimize or eliminate the observed differences between human and chimp kinases.

**Conclusion:**

None of the proposed novel chimpanzee kinase architectures are supported by experiment evidence. Guidelines to prevent such erroneous conclusions in similar papers are proposed.

## Background

In a recent paper in this journal, Anamika *et al *present an analysis comparing the sets of protein kinases encoded in the human and chimpanzee genome. Based on this, they concluded that chimps possess many kinases with unprecedented domain structures.

Such a finding is highly surprising, given the very close similarity of chimpanzees and humans. The overall nucleotide identity between the two genomes is around 95%, with much of the difference found in the form of small insertions in one genome relative to the other [2]. Indeed, humans have been nicknamed "The Third Chimpanzee" [3].

A re-analysis of the results of Anamika *et al *shows that several of their reported novel structures are entirely known. Furthermore, their results consistently invest in the chimp gene models a degree of confidence which they do not warrant, given that there is very little EST data for chimps and so most models are *de novo *predictions. In many cases, sequence data available prior to the submission of the Anamika *et al *paper supplies alternate, more conservative interpretations of chimp gene structure. Two examples are examined in depth below, but this excessive interpretation of questionable gene models pervades the Anamika *et al *work.

## Results & Discussion

**Figure 1:**
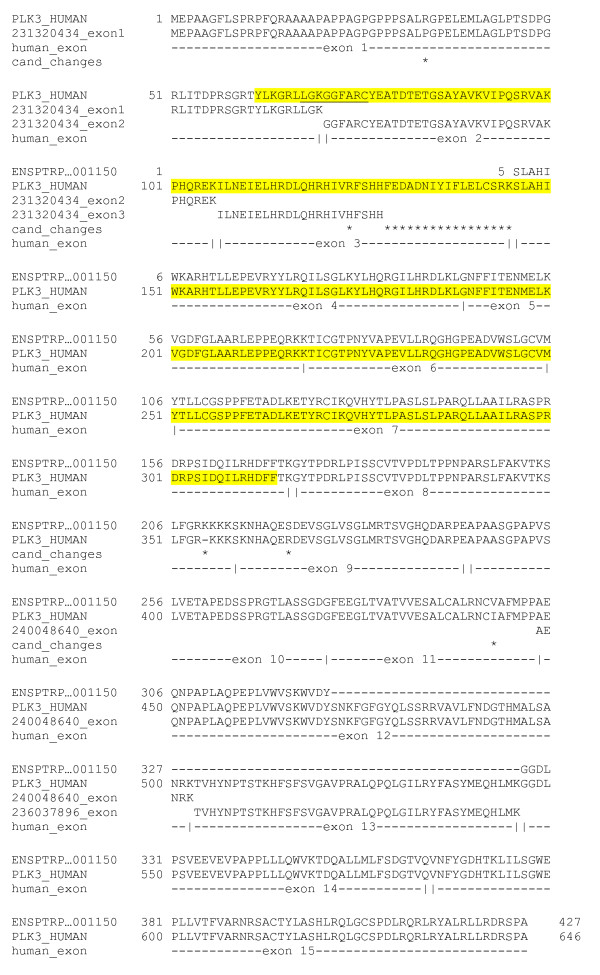
**Alignment of ENSPTRP00000001150 with PLK3 from human (RefSeq NP_004064.2), exon structure from human, and chimp Whole Genome Shotgun reads from the NCBI trace archive corresponding to segments homologous to human PLK3 but missing from ENSPTRP00000001150**. Asterisks mark remaining amino acid changes between human and chimp PLK3 if all of the additional information is incorporated. Exons implied by chimp whole genome shotgun traces (NCBI Trace Archive entries 231320434, 240048640 and 236037896) are also shown. The kinase domain of human PLK3 is highlighted in yellow, with the key ATP binding region underlined.

In one case, Anamika *et al *claim to identify a chimp kinase (ENSPTRP00000001150) whose closest kinase domain relative in human has 31% identity, a distance which is nearly unimaginable given the great similarity between chimps and humans. Furthermore, this particular kinase is claimed to have greatest similarity to casein kinase 1 but possesses a polo box, a domain involved in the specific recognition of phosphorylated peptides. Polo boxes have been found only in polo-like kinases [4], and so to find a polo box on a kinase in a different subfamily (such as casein kinase 1) would be a very remarkable finding. However, a BLAST search of the chimp ORF against the RefSeq human protein database reveals the best human match to be Polo-like kinase 3 (PLK3), with >90% sequence identity overall (Figure 1) and 100% identity. Furthermore, careful searching of the chimpanzee whole-genome shotgun sequence reveals reads consistent with most of the pieces missing relative to human PLK3 (Figure 1), with the exception of a 3' portion of exon 3. Supplementation with this additional data yields a chimp gene model with 100% identity in the protein kinase domain (positions 62 to 314 as annotated in UniProt). The restored sequence also contains the ATP-binding site, as annotated in UniProt (positions 68-76); hence the chimp gene model used by Anamika et al is either incomplete or non-functional as a kinase due to the essentiality of this site to protein kinase function. While it cannot be conclusively demonstrated that these should be incorporated into the chimp gene model, their presence in the raw sequence data suggests that a finished assembly would probably contain the missing exonic regions. It is also worth noting that the missing pieces each correspond to one or more contiguous exons; in other words the differences between the chimp model and the human protein are entirely explainable by the gene prediction program skipping exons. One interesting possibility raised by these chimp fragments is that chimp PLK3 has deleted a short region of exon 3. This is supported by two reads in the NCBI Trace Archive. However, given the sparseness of data it could also be the case that the remainder of exon 3 is present in the chimp genome but as yet unsequenced or that both of these reads contain artifacts preventing the detection of the missing portion. Furthermore, the chimp protein contains an insertion (GGDLPSVEEVEPAPP) relative to both human and macaque proteins. Otherwise, it is striking that the potential chimp exons have precisely the same amino acid boundaries as the known human gene structure. In any case, the simplest conclusion is that ENSPTRP00000001150 is chimp PLK3 and its possession of a polo-box therefore unsurprising.

The treatment of ENSPTRP00000001150 exemplifies several analytic practices in the Anamika paper which lend themselves to error. First, the paper's methodology uses Ensembl as the source of the human proteins. Ensembl is a powerful genomic analytic tool, but it relies on automated protein predictions and the proteins lack authoritative assignment to genes. The RefSeq protein database would have been a far superior reference database (as implied by the name!). Comparing against the full non-redundant protein database is also a useful approach to test conclusions made using the highly annotated, but incomplete, RefSeq database. The tree purporting to show the relationship of ENSPTRP00000001150 to casein kinases should have served as a warning flag as well, for the chimp kinase cannot be ruled out as an outgroup in this figure. Ensuring the rooting of the casein kinase subfamily by including representative members of other kinase subfamilies might have flagged this error. Even better, a paper reporting a pairwise comparison of one proteome subset with another would be well served to present that entire alignment score matrix as a supplementary table. A paper such as this should also include all of the protein kinase sequences used as a supplementary file so that its conclusions can be rapidly validated by reviewers and interested readers.

**Figure 2:**
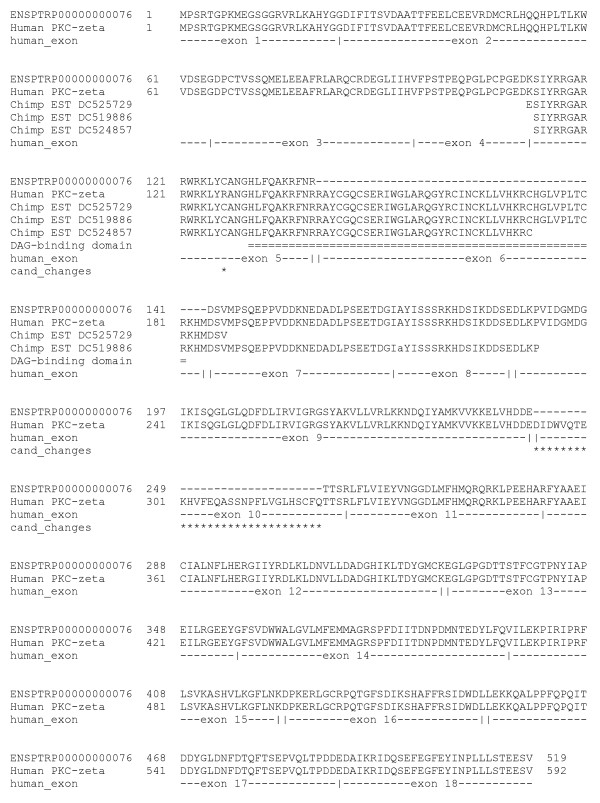
**Alignment of ENSPTRP00000000076, human PKC-zeta isoform 1 (RefSeq NP_002735.3) and chimp ESTs containing sequences homologous to human PKC-zeta but missing from ENSPTRP00000000076**. The lowercase 'a' for DC519886 requires the insertion of 1 nucleotide to maintain frame. Asterisks mark remaining amino acid changes between human and chimp PLK3 if all of the additional information is incorporated.

The chimp protein ENSPTRP00000000076 is claimed as a "chimpanzee specific multi-domain architecture" containing a PB1 domain followed by a protein kinase C-like domain. This particular pairing is then claimed to have been found only in a single sea squirt kinase. Re-alignment to RefSeq reveals that the domain architecture was correctly described here. However, this kinase is clearly the chimp ortholog of human PKC zeta with 87% identity (Figure 2). Long before the chimpanzee genome was sequenced, two different groups described this architecture in human PKC and demonstrated the formation of dimers via the PB1 domain [5,6]. ENSPTRP00000000076 is also claimed to lack a diacylglycerol (DAG) binding domain, but three chimpanzee ESTs deposited in October 2007 (DC524857, DC519886 and CD524857) are consistent with the presence of the DAG-binding domain in chimpanzee PKC-zeta (Figure 2). The dropped region corresponds precisely to the sixth exon of human PKC-zeta isoform 1.

These flaws are repeated in several of their other examples of "novel" chimpanzee architectures where the chimp sequence contains insertions or deletions relative to human. In no case does the evidence for the novel chimp architecture go beyond a *de novo *gene prediction purely from genomic DNA. Such prediction is well-recognized as a difficult and error-prone challenge, and in the absence of more direct evidence (such as EST support) such predictions cannot be trusted. One recent paper did identify numerous indels between chimp and human using the same sort of sequence data [7]. However this paper verified these changes via experimental PCR evidence; such validation is lacking in the Anamika paper.

Presenting bioinformatics results in a manner which is accessible both to experts in the field can be challenging. In particular, the appropriate way to identify sequences presents tradeoffs: unique accession numbers eliminate ambiguity for computational biologists, but make it impossible for a biologist to quickly assess the biological plausibility or implications of a result. The Anamika et al paper illustrates the critical importance of presenting results in a biologically legible manner, so that surprising findings can be quickly cross-checked. This is particularly relevant in the kinase field, where the elegant kinase tree of Manning *et *[8] has become iconic and appears on posters, apparel and as a basis for figures in other papers (e.g. Karaman et al [9]). As noted above, with the inclusion of proper supplementary files cross-referencing the meaningful identifiers to accessions, the risk of ambiguity is eliminated.

One final recommendation is made for the design of such computational analyses. As useful as a correctly executed pairwise comparison to human could be, even more valuable would be a three-way comparison with mouse. The mouse genome has been finished to a similar quality standard as the human genome, and an in depth analysis of its protein kinases has been published [10]. Such a third reference would be useful for putting any unusual architectures into context, and would also greatly reduced the risks of mis-identifying chimp PLK3 as a casein kinase.

## Concluding remarks

None of the domain architectures proposed by Anamika *et al *appear to be both novel and well-supported; the few that are well supported have been previously described in the literature and the remaining novel architectures lack any experimental support and are therefore likely to be the artifacts of *de novo *gene prediction on a draft quality genome. In many cases, bringing in additional evidence in the form of chimp ESTs or individual WGS reads undermines the architectures analyzed by Anamika *et al*. These sequences appear to have been available at the time of their analysis and therefore could have been consulted as part of the totality of evidence for (or against) the gene structures of chimpanzee protein kinases. More careful choices of reference proteomes would likely have prevented many of the misidentifications, and more careful attention to the quality of the evidence for the conclusions drawn would have led to them being carefully hedged, if made at all.

## Authors' contributions

### 

KR conceived and executed all parts of this project. No external funding was utilized.

## **Response to the article *Re-examination of chimp protein kinases suggests"novel architectures" are gene prediction artifacts *by K. Robison**

Krishanpal Anamika^1^, Juliette Martin^2^, Suruchi Bakshi^3 ^& Narayanaswamy Srinivasan

Molecular Biophysics Unit, Indian Institute of Science, Bangalore 560012, India

***Current address***: ^1^Department of Functional Genomics, Institute of Genetics and Molecular and Cellular Biology, 1 rue Laurent Fries/BP 10142/67404 Illkirch Cedex

Strasbourg, France. ^2^Universit'e de Lyon, Lyon, France; Universit'e Lyon 1; IFR 128; CNRS, UMR 5086; IBCP, Institut de Biologie et Chimie des Prot'eines, 7 passage du Vercors, Lyon, F-69367, France. ^3^Doctoral Training Centre, University of Oxford, Wolfson Building, Oxford, OX1 3QD, UK

The article by Robison [11] discusses an important point on the validity of gene prediction for the presently available genome assembly of chimpanzee genome. The entire discussion is unfortunately misplaced in the context of analysis of domain combinations in protein kinases encoded in the chimpanzee genome that was reported by Anamika et al [12]. In general, roots of such problems stem not only from the quality of gene prediction but also from the quality of the genomic data and assembly. Though the discussion is misplaced within a highly specific context of domain combinations in chimpanzee kinases it is certainly important to discuss and understand the quality of genomic assembly of chimpanzee and the quality of gene predictions as they could have significant impact on the results of any analysis on chimpanzee proteome.

Anamika et al [12] have used the ENSEMBL database (release 46) in their comparative analysis of human and chimpanzee kinases. In our opinion, ENSEMBL has set quite high standards on the quality of datasets disseminated including gene annotation [13]. High quality of the datasets is achieved in ENSEMBL in a series of stages. Of particular relevance to the comparison of human and chimpanzee proteomes, we would like to draw the attention to the steps employed by ENSEMBL: In the first stage of the gene build species-specific proteins are aligned to the genome and a transcript structure is built for the protein on the genome. It must be noted that this step is followed by the similarity stage, in which proteins from closely related species are used to build transcript structure in regions were a targeted transcript structure is absent. For those species having significant number of experimentally identified protein sequences, this stage has a major impact on the gene structures in the genebuild. However, according to the documentation of ENSEMBL, for species with fewer species-specific protein sequences, this stage plays an even more important role in predicting gene structures. It should also be noted that the next stage in ENSEMBL involves alignment of species-specific cDNA and EST sequences to the genome and a careful treatment of non-translated region of cDNA. The final set of prediction corresponds to multi-transcript genes as it is derived by combining identical transcripts corresponding to different protein sequences. For every transcript model, the protein and mRNA sequences serve as supportive evidence.

Based on these steps taken in ENSEMBL, it is clear that automatically annotated genes too have strong mRNA and cDNA evidence from other species. Thus it is difficult to accept that there are serious problems with many of the genes predicted and listed in ENSEMBL database.

Robison points out differences between ENSEMBL and RefSeq databases in terms of annotation and gene prediction. While this matter is certainly beyond the analysis of domain combinations in kinases, it is more a comparison of quality of two different databases which are of high quality in their own right. As stated by Anamika et al in their paper [12], the human proteome data used in their analysis is based on the NCBI 36 assembly of the human genome, released in November 2005. It is composed of 43,570 protein sequences: 3,614 "novel peptides", 18,642 "known peptides" and 21,314 "known-ccds peptides". The annotation "known-ccds" suggests that these sequences are part of a core set consistently annotated, high quality data in the frame of the Consensus Coding Sequence (CCDS) project. The phrase "known peptides" suggests that these proteins were mapped to entries in Swiss-Prot, RefSeq or SPTrEMBL during the annotation process. Given the high proportion of proteins that are flagged as "known-ccds" or "known peptides", we are of the impression that the quality of the data used by Anamika et al is generally very good.

Although we believe that ENSEMBL dataset is of high quality, we agree with Robison that, in general, quality of the genome sequence and assembly as well as quality of gene prediction can suggest dubious dissimilarities between chimpanzee and human proteins. However, based on our more detailed comparative analysis of entire proteomes of human and chimpanzee there are genuine significant differences between proteins of human and chimpanzee that can not be easily explained on the basis of genome sequence quality and gene prediction. For example, we consider the cases discussed by Robison in the later sections of this article.

Unfortunately there are a few points of comprehension, different from what Anamika et al intended, expressed in Robison article. For example, Robison states: "......they (Anamika et al) concluded that chimps possess many kinases with unprecedented domain structures". It should be noted that Anamika et al report 587 putative protein kinases in chimp and they report only 3 cases of chimp-specific architectures, displayed in Figure three a and discussed in the text. We believe that Robison's contention of 3 out of 587 (0.5%) as "many" is inappropriate. On the contrary 584 chimp kinases do not exhibit any difference in terms of domain structure compared to human kinases.

In another case Robison seems to have understood that Anamika et al claim to identify a chimp kinase (ENSPTRP00000001150) whose closest relative in human has 31% identity. Anamika et al did not imply such a point. Anamika et al did not state that when searching for orthologues of ENSPTRP00000001150 in the human proteome, the closest human sequence is found to have 31% sequence identity. Indeed in Table two of the Anamika et al paper, the closest human orthologue of ENSPTRP00000001150 is listed as ENSP00000361275 which is described as PLK3_HUMAN shown on Figure one in Robison paper. The difference between sequence identity reported by Robison (>90%) and us (65.5%) comes from the formula used to compute the percentage: with respect to the length of the sequence or the length of the alignment. Thus, in this example the apparent contradiction stems from difference in understanding. Indeed, both Anamika et al and Robison concur on the matter of nearest human protein of ENSPTRP00000001150.

Robison provides an impression that Anamika et al considers this kinase as a casein kinase 1 with a polo box. Again, Anamika et al did not mean to give such an impression. On the contrary Anamika et al state in page 3 of their paper that the catalytic domain of the sequence ENSPTRP00000001150 was classified as casein kinase 1 subfamily because it has 31% identity and e-value equal to 2 × 10^-16 ^with the Position Specific Scoring Matrix (PSSM) of catalytic domain of casein kinase 1 in the Reverse BLAST analysis. In no way it means that the closest relative in human is 31% identical. However the point made by Anamika et al, but, seemed to have missed by Robison is that the amino acid sequence pattern of the catalytic region of ENSPTRP00000001150 is similar to casein kinase 1 PSSM. As stated in our paper, we have used multiple PSSMs created for the 55 protein kinase subfamilies represented in Hanks and Hunter classification scheme [13] as query to search against the chimpanzee proteome using RPS-BLAST. The kinase domain of ENSPTRP00000001150 has shown to have highest similarity with a PSSM representative of the casein kinase 1 subfamily. So, this is an interesting case of kinase catalytic region more similar to casein kinase 1 while the full length protein sequence is more similar to that of a POLO kinase due to the occurrence of a POLO box in ENSPTRP00000001150. So, we contend that this is a difficult case of classification purely from sequence analysis. It will be interesting to understand the role of ENSPTRP00000001150 in signaling process from experimental studies when they become available.

Robison provides an interesting analysis of whole-genome shotgun sequence comparing the genomic regions of ENSPTRP00000001150 and ENSP00000361275 and speculates that the finished product will probably contain currently missing exonic regions. We concur with this speculation. This is a good possibility to look for when genomic data with better accuracy becomes available. However currently it remains speculative. Robison identified the ATP-binding site in human ENSP00000361275/PLK3_HUMAN, which is missing in the chimp sequence. Robison thus concludes that this chimp protein is either incomplete or non-functional. We agree that the corresponding protein, if complete and shown to lack ATP binding site, might be non-functional as a kinase. It should be emphasized that Anamika et al discussed this example as chimp protein kinases with interesting difference compared to their closest human homologue.

Anamika et al reported that the example of the chimp kinase ENSPTRP00000000076 contains a PB1 domain followed by a protein kinase C-like domain and they further reported that this domain architecture is seen only in a sea squirt kinase. Although Anamika et al used Pfam database version 22.0, the architecture PB1/catalytic domain/PKC terminal domain is actually reported only in a single sea squirt kinase in Pfam version 23.0. Kinases discussed in references 5 and 6 of Robison paper possess a DAG binding domain between PB1 and catalytic domains. Anamika et al also identified these cases in their analysis. In page 7 of the Anamika et al paper it is stated that "Our analysis identified two chimpanzee PKCs and a human PKC with a similar architecture, in which a phorbol esters/diacylglycerol binding domain is inserted between the PB1 and the protein kinase domain." The Robison article reports that three chimpanzee ESTs deposited in October 2007 (DC524857, DC519886 and CD524857) are consistent with the presence of the DAG-binding domain in chimpanzee PKC-zeta. In this context it should be pointed out that Anamika et al report has identified the PB1/DAG binding/catalytic domain/PKC terminal domain architecture in two chimp kinases. Indeed in Table two of Anamika et al the authors have already mentioned ENSP00000367830 (human PKCzeta) as 87% identical to the chimp kinase ENSPTRP00000000076 that was also mentioned by Robison as the point missed by Anamika et al. Robison provides an interesting analysis of ENSPTRP00000000076 and human PKC-zeta with exonic structure, showing that the deletion in ENSPTRP00000000076 corresponds precisely to the sixth exon of human PKC-zeta isoform 1, and concludes that sequence of ENSPTRP00000000076 is incomplete. This remains to be confirmed when better genomic data becomes available.

So, we disagree with the statement made by Robison that none of the domain architectures proposed by Anamika et al appear to be both novel and well-supported. Indeed many of the points raised by Robison seem to stem from misunderstanding of the statements made by Anamika et al. We agree with Robison who suggests that a list of all the kinase sequences analyzed should be made available publicly. Indeed all the sequences used in the Anamika et al analysis are publicly available in the KinG web site http://hodgkin.mbu.iisc.ernet.in/~king as mentioned in their paper.

In the context of discussion on how similar a human protein sequence could be to the nearest homologue in chimpanzee Robison appropriately recalls very high nucleotide sequence identity between the two genomes. We agree with this point. However, it has also been pointed out by the Chimpanzee Genome Sequencing and Analysis Consortium [14] while comparing chimpanzee and human genomic data that "Insertion and deletion (indel) events are fewer in number than single-nucleotide substitutions, but result in ~1.5% of the euchromatic sequence in each species being lineage-specific". Also, it is not clear if the possibilities of recombination/horizontal gene transfer/insertion-deletion/frameshift events in either of the genomes can be precluded.

Finally, chimpanzee proteome has been generated from an automatic annotation system based on biological evidences [14]. The human genome data, which is of relatively high quality, was used as a guide for annotation, by projecting the human gene models onto the chimpanzee genome. Further, ENSEMBL carefully determines transcript on a case-by-case basis

(see http://www.ensembl.org/info/docs/genebuild/genome_annotation.html) [15]. It should be noted that all ENSEMBL transcripts are also based on experimental evidence from EMBL, UniProtKB, and RefSeq. Clearly, one cannot do better than the data one is working with. We contend that, in general, chimpanzee proteome dataset in ENSEMBL is of good quality and not all the differences one notes between human and chimpanzee proteins can be explained solely on the basis of quality of gene prediction. It is premature to conclude so at this stage. While Anamika et al focused only on differences in domain combinations of human and chimpanzee kinases, even more significantly, a recent analysis [16] identified a few completely unique genes in human compared to chimpanzee by a very careful analysis which provides useful guidelines on analysis of chimpanzee genomic data. Also, as pointed out by Robison, the work of Volfovsky et al [17] reports numerous indels, which are experimentally validated, in the sequence data of chimp and human. Work of Knowles and McLysaght [16] and Volfovsky et al [17] suggests that more concrete conclusions can only be arrived at when such experimental validation has been achieved in a much larger scale.
